# Theoretical analysis on thermal treatment of skin with repetitive pulses

**DOI:** 10.1038/s41598-021-89395-x

**Published:** 2021-05-11

**Authors:** Jingxuan Ma, Xianfeng Yang, Yuxin Sun, Jialing Yang

**Affiliations:** School of Aeronautic Science and Engineering, Beihang University, Beijing, 100191 People’s Republic of China

**Keywords:** Biomedical engineering, Computational science

## Abstract

Thermal ablation is an efficient method of medical treatment, such as cancer therapy, wound closure, laser cutting, freckle removal and other treatments. In order to guarantee the curative effect and the safety of the patients, the thermal response of the tissue which is subjected to the heat source need to be carefully monitored. However, it is too difficult to achieve real-time monitoring on the full-field temperature. In the present study, efforts were made to build up a theoretical model for the prediction of the thermal response in the human skin. The Dual-Phase-Lag (DPL) bio-heat transfer model and the Henrique’s burn assessment model were employed to describe the interaction of multi-pulse heat source and the skin. The repeated multi-pulse laser is a common heat source in the thermal treatment and the thermal responses of the skin would be complicated under the common effects of the non-Fourier effects and the multi-pulse source. The Green’s function approach was used to solve the governing equations analytically. The closed-form solution for the temperature distribution of the skin was obtained and the thermal damage was estimated based on the temperature results. The influences of the biological parameters (the phase lags of the heat flux and the temperature gradient) and the heat source parameters (the pulse number and the duty ratio) on the temperature distribution, the burn degree and the irreversible burn depth of the irradiated region were discussed.

## Introduction

Thermal ablation is an efficient method of medical treatment, such as cancer therapy, wound closure, laser cutting, freckle removal and other treatments. In thermal ablation, human tissues are exposed to different kinds of heat producers, including microwave, radio-frequency, infrared radiation, magnetically excitable thermo-seeds and other thermal sources^1^. However, the high temperature could hurt the healthy cells by loosening the cell membranes and denaturing the proteins^2^. In order to guarantee the curative effects and the patients’ safety, the temperature distribution in human tissue should be strictly controlled. Given that real-time monitoring on the full-field temperature cannot be achieved at present, it is extremely significant to make precise prediction of the temperature distribution before thermal treatment. The mechanism of the thermal responses in human tissue during the hyperthermia need to be investigated in detail.

Among the existing literatures, Pennes^3^ was the first to study the heat conduction in the living biological tissues by proposing a bio-heat transfer model on the basis of Fourier’s law. Since then, the Pennes model has been widely applied by researchers. For example, Yue^4^ developed a one-dimensional steady-state bioheat transfer model of living tissues in cylindrical coordinates and derived the analytical solution with the usage of Bessel’s equation. Shih^5^ investigated the thermal response of a semi-infinite biological tissue due to a sinusoidal heat flux at the skin. Laplace transform method was employed to obtain the semi-analytical solution for the governing equations. Fu^6^ explored the thermal response inside the skin in the process of thermal treatment on the basis of Pennes model. The influences of the tumor shape, including the location, geometry and size, on the temperature distribution were taken into account. Singh^7^ investigated the thermal effects on the breast tumor during the radio-frequency ablation. The effects of temperature-dependent parameters of the multi-layer breast model, such as electrical and thermal conductivities, were considered. Bhowmik^8^ carried out a numerical study on the thermal response of skin with different fat thickness. The Pennes model and the numerical simulation method were employed to derive the temperature distribution of the skin tissue. Marchandise^9^ probed the feedback from human nociceptive system on the thermal stimuli with finite element simulation and Fourier’s law employed. The influences of the water-content rate in both hairy skin and glabrous skin were discussed emphatically.

All the above researches were based on the traditional Fourier’s law which assumed that thermal propagation velocity is infinite. However, this assumption was found not suitable for some issues in which the non-Fourier effects shows up, such as heat transfer problems in low-temperature environment and in the biological tissue^10–12^. To make the thermal conduction model more accurate, Cattaneo^13^ and Vernotte^14^ established a thermal wave model (C-V model) with a finite thermal propagation velocity by involving the heat flux relaxation time *τ*_*q*_. Ulteriorly, Tzou^15^ promoted this heat transfer model by introducing another relaxation time *τ*_*T*_ for the phase lag of temperature gradient. The improved model is called dual-phase-lag (DPL) bio-heat conduction model which allows either the temperature gradient to precede heat flux vector or the heat flux vector to precede temperature gradient. Researchers^16,17^ investigated the heat transfer problems in spherical coordinate system with DPL model and numerical method employed. The influences of the non-Fourier effects induced by the phase lags of the heat flux and the temperature gradient on the thermal behavior were considered. Afrin^18^ explored the non-equilibrium heat transfer in living biological tissues by considering the arterial and venous bloods. They found that the phase lags of the heat flux and the temperature gradient depend on the properties of artery, vein and tissue, blood perfusion rate and convective heat transfer rate. Nobrega^19^ studied the heat conduction in the skin subjected to the pulse laser heating and fluid cooling. The analytical solutions for the one-dimensional skin model were presented with three different heat conduction models employed. Jaunich^20^ analyzed the temperature distributions in the skin which is subjected to a short pulse laser. The results from the hyperbolic model and the parabolic model were compared.

It is of great significance to explore the mechanism of thermal damage to make sure of the reliability and safety of thermal treatments. For this purpose, Henriques^21^ proposed an expression for the protein denaturation process based on the first order approximation of the Arrhenius equation, which has been widely used in the burn degree prediction. Lu and co-authors^22,23^ developed a computational approach to examine the heat transfer process, heat-induced mechanical response, as well as the associated pain level with numerical simulation and the finite difference method employed. Liu^24^ analyzed the thermal response for estimating thermal damage in laser irradiated biological tissue with DPL model employed. Afrin^25^ investigated the thermal damage in the living biological tissue induced by laser irradiation with a generalized DPL model employed. The results derived from the generalized and classical DPL model were compared and the influences of the laser parameters were discussed. Wang^26^ investigated the thermal response of the skin which is subjected to the pulse boundary heat flux with Laplace transform technique employed. Venugopalan^27^ explored the thermodynamic response of biological tissue by experimental and analytical methods.

The complication of the transient heat conduction equations with non-Fourier effects under consideration makes the theoretical results very difficult to derive. Green’s function method can be beneficial to solve the complex differential equations of mathematical physics. Zur^28^ investigated the free vibrations of functionally graded circular plates with elastic supports on the basis of the classical plate theory. Green’s function method was employed to solve the boundary value problem. Chao^29^ presented the thermoelastic behavior of two circular inclusions in an infinite plane which is subjected to a point heat source. The solution obtained in the study can be treated as the Green’s functions for the crack problem associated with two circular inclusions. Seremet^30,31^ inspected the steady-state thermoelastic problems for domains described in cylindrical coordinate system and Cartesian coordinate system. A new method was proposed to derive the Green’s function for incompressible Lamé equations and the Green’s function method was used to solve the governing equations. Zhao^32^ investigated the coupled thermoelastic vibration of the Euler–Bernoulli beam with cracks which was subjected to heat flux. The explicit expressions of the temperature and displacement response in the beam were obtained with the usage of Green’s function method. Ma^33^ has solved some heat transfer problems by using Green’s function method, such as the three-dimensional living biological tissue subjected to a scanning laser beam, the DPL heat conduction in a finite medium subjected to a moving feat source, and the bi-layered circular plate irradiated by laser pulse.

In clinical medicine, the continuous laser or pulsed laser is selected for different disease. In addition, it is reported that both single pulse laser and repetitive pulse laser can be used^32^. At present, the thermal response of human tissue under continuous laser or single pulse laser has been investigated widely. However, there is few reports about the response induced by repetitive laser. In this case, the thermal conduction equations should be solved respectively in different time segmentations because of the discontinuity of the thermal source, which makes it difficult to predict the temperature distribution during the thermal ablation. To address this issue, the present study will focus on the heat transfer conduction procedure in a three-dimensional model in vivo skin which is subjected to a repetitive pulse laser. The governing equations will be established on the basis of DPL model. Exact solution of temperature distribution will be obtained by employing the Green’s function method, which is meaningful for the further exploration on the physical mechanism of the thermal treatment. The thermal damage will be evaluated and the influences of non-Fourier effects will be discussed.

## Mathematical models

The heat conduction model employed in the present study is the DPL model, which is modified from the Fourier’s law with two relaxation times under consideration. This heat conduction model can be expressed as^34^:

1 $$ q\left( {\overrightarrow {r} ,t + \tau_{q} } \right) = - k\nabla T\left( {\overrightarrow {r} ,t + \tau_{T} } \right) $$

where, *τ*_*q*_ and *τ*_*T*_ represent the phase lags of the heat flux and the temperature gradient, *q* the heat flux, *k* the heat conductivity, *T* the temperature, $\overrightarrow {r}$ and *t* the spatial and temporal coordinates, respectively.

The thermal conduction properties of the skin are quite different from that in the classic homogeneous materials. The thermal response will be influenced by the convection between blood and the skin, blood perfusion in vascular beds and metabolic heat generation. Pennes^3^ considered the influences as an average distribution in the biological tissue and established a bio-heat conduction model (Pennes model) which has been widely used for decades of years. With the combination of the Pennes model and the DPL model^15^, proposed a modified bio-heat transfer model, which can be written as:

2 $$ \begin{gathered} \rho C\tau_{q} \frac{{\partial^{2} T}}{{\partial t^{2} }} + \left( {\rho C + \tau_{q} w_{b} \rho_{b} C_{b} } \right)\frac{\partial T}{{\partial t}} = \hfill \\ k\nabla^{2} T + k\tau_{T} \frac{\partial }{\partial t}\left( {\nabla^{2} T} \right) + w_{b} \rho_{b} C_{b} \left( {T_{a} - T} \right) + \tau_{q} \left( {\frac{{\partial Q_{m} }}{\partial t} + \frac{\partial Q}{{\partial t}}} \right) + Q_{m} + Q \hfill \\ \end{gathered} $$

where, *ρ* and *C* are the density and specific heat of the skin. *ρ*_*b*_, *C*_*b*_, *w*_*b*_ represent the density, specific heat and perfusion of blood, respectively. *T*_*a*_ is the temperature of arterial blood, *Q*_*m*_ for the metabolic heat generation and *Q* for the external heat source.

**Figure 1:**
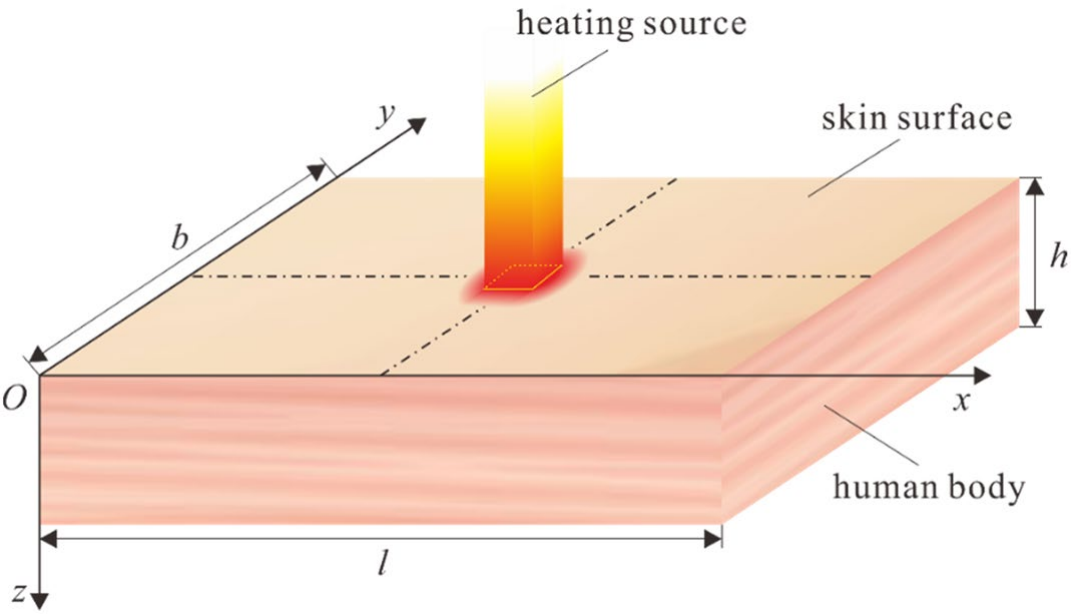
Illustration of the 3D skin model.

In the present study, the thermal transfer process in a cuboid skin model which is subjected to laser pulses is investigated. As is shown in Fig. 1, the length, width and height of the model are *l*, *b* and *h*, respectively. The rectangular laser pulses irradiate the cuboid at the center of the top surface. The governing equation on the basis of DPL bio-heat conduction model can be expressed as:

3 $$ \begin{gathered} \rho C\tau_{q} \frac{{\partial^{2} \theta }}{{\partial t^{2} }} + \left( {\rho C + \tau_{q} w_{b} \rho_{b} C_{b} } \right)\frac{\partial \theta }{{\partial t}} = \hfill \\ k\nabla^{2} \theta + k\tau_{T} \frac{\partial }{\partial t}\left( {\nabla^{2} \theta } \right) - w_{b} \rho_{b} C_{b} \theta + \tau_{q} \left( {\frac{{\partial Q_{m} }}{\partial t} + \frac{{\partial Q_{l} }}{\partial t}} \right) + Q_{m} + Q_{l} \hfill \\ \end{gathered} $$

where, *θ*  = *T* *–* *T*_*0*_ is the temperature rise in the skin, *T*_*0*_ for the core temperature of human body. *Q*_*l*_ represents the power density of the laser source, which can be written as:

4 $$ Q_{l} = \varphi \left( z \right)Q_{1} \left( x \right)Q_{2} \left( y \right)Q_{3} \left( t \right) $$

*φ*(*z*), *Q*_*1*_(*x*), *Q*_*2*_(*y*) and *Q*_*3*_(*t*) are expressions for the spatial and the temporal distributions of the laser energy, which are given by

5 $$ \varphi \left( z \right) = \left( {1 - R_{a} } \right)I_{0} \mu_{a} \exp \left( { - \mu_{a} z} \right) $$

6 $$ Q_{1} \left( x \right) = H\left[ {x - \left( {\frac{l}{2} - \frac{{R_{0} }}{2}} \right)} \right] - H\left[ {x - \left( {\frac{l}{2} + \frac{{R_{0} }}{2}} \right)} \right] $$

7 $$ Q_{2} \left( y \right) = H\left[ {y - \left( {\frac{b}{2} - \frac{{R_{0} }}{2}} \right)} \right] - H\left[ {y - \left( {\frac{b}{2} + \frac{{R_{0} }}{2}} \right)} \right] $$

8 $$ Q_{3} \left( t \right) = \sum\limits_{i = 1}^{{n_{pulse} }} {\left[ {H\left( {t - t_{o}^{\left( i \right)} } \right) - H\left( {t - t_{e}^{\left( i \right)} } \right)} \right]} $$

where, *µ*_*a*_ is the absorption coefficient of the skin, *H*(*) for the Heaviside function, *R*_*a*_ for the optical reflectivity, *I*_*0*_ for the power density of the input laser energy, *R*_*0*_ for the geometric dimension of the laser spot. *t*_o_^(*i*)^ and *t*_e_^(*i*)^ are the onset moment and the ending moment of the *i*th pulse and *n*_*pulse*_ is the number of the pulse.

The model is considered as a cuboid domain in human body. So temperatures of the side surfaces and the bottom surface are assumed equal to the core temperature *T*_*0*_. The top surface is considered as the skin so the natural convection between the top surface and the air is under consideration. The boundary conditions can be expressed as:

9 $$ \left\{ \begin{gathered} \left. \theta \right|_{x = 0} = \left. \theta \right|_{x = l} = 0 \hfill \\ \left. \theta \right|_{y = 0} = \left. \theta \right|_{y = b} = 0 \hfill \\ \left. \theta \right|_{z = h} = 0 \hfill \\ \left. {\left( { - k\frac{\partial \theta }{{\partial z}} + h_{1} \theta } \right)} \right|_{z = 0} = f_{1} \hfill \\ \end{gathered} \right. $$

where, *f*_*1*_ = − *h*_*1*_ (*T*_*0*_ *−* *T*_*e*_). *T*_*e*_ is the ambient temperature and *h*_*1*_ is the convective heat transfer coefficient.

The initial conditions can be expressed as:

10 $$ \left\{ {\begin{array}{*{20}c} {\left. \theta \right|_{t = 0} = 0} \\ {\left. {\frac{\partial \theta }{{\partial t}}} \right|_{t = 0} = 0} \\ \end{array} } \right. $$

**Figure 2:**
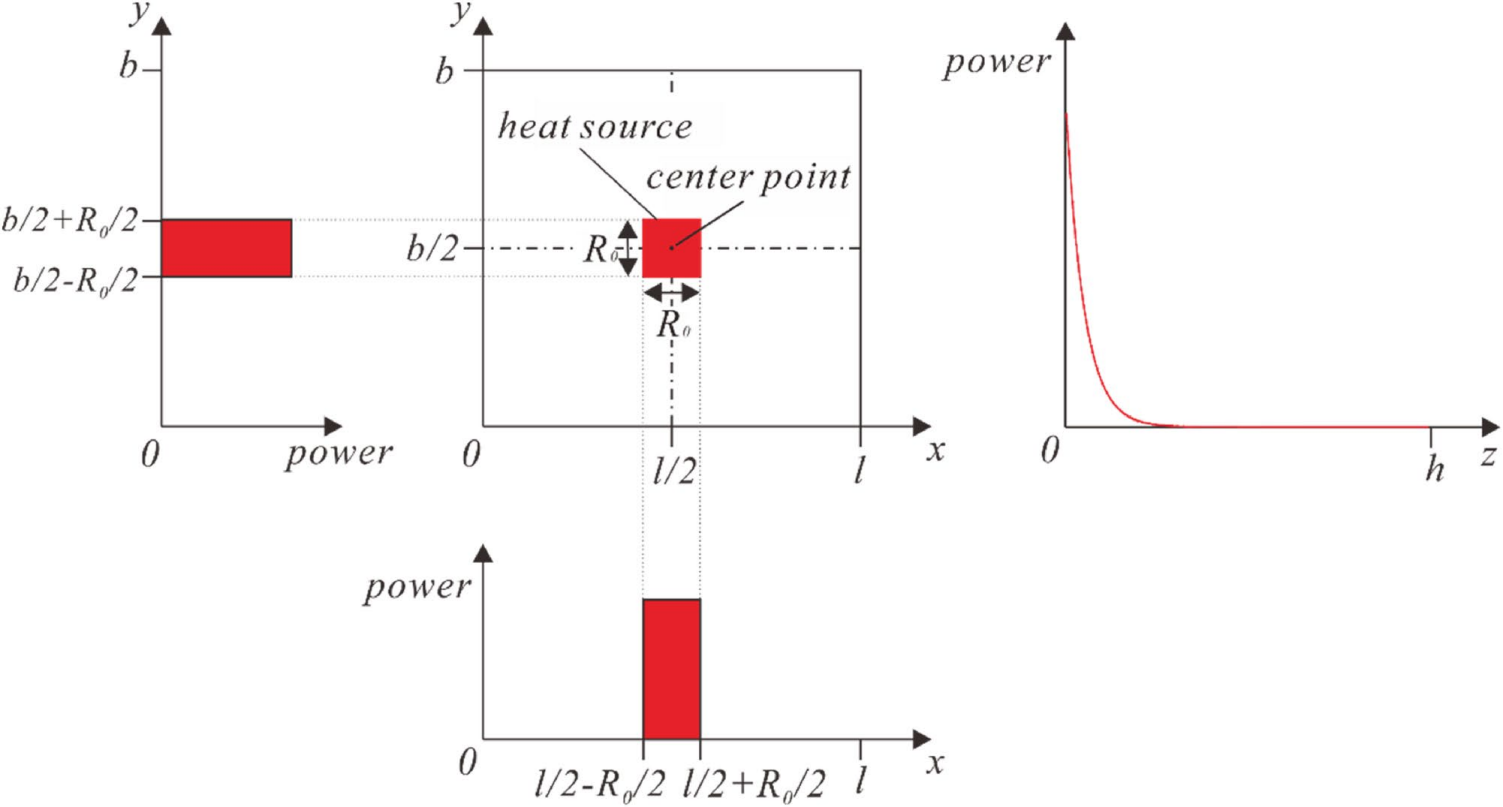
Spatial distribution of the heat source power.

**Figure 3:**
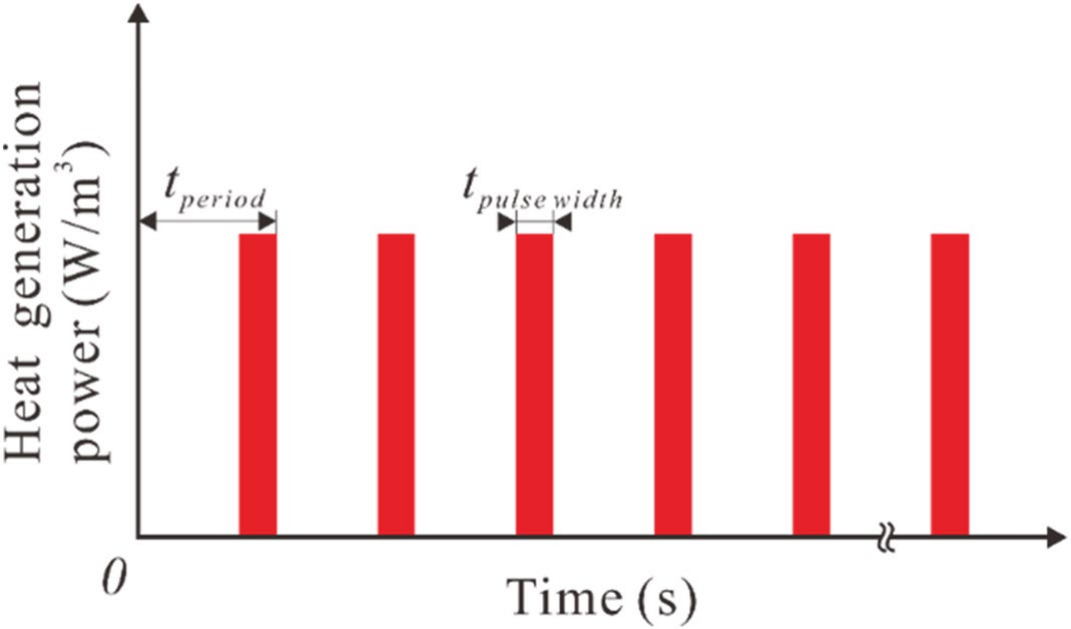
Temporal distribution of the heat source power.

The laser pulse which irradiates the skin is treated as the volume heat generation in the substrate. Figure 2 shows the spatial distribution of the input energy power. The energy input is in the exponential decay distribution along depth direction. On the top surface of the skin, the heating energy evenly distributes in a square area. In the time domain, the laser energy is distributed in the form of repeated multi-pulse, as is shown in Fig. 3. It is assumed that the period of each laser pulse is *t*_*period*_ with the duration time being *t*_*pulse-width*_. It is clear that *t*_*pulse-width*_ = *t*_o_^(*i*)^ − *t*_e_^(*i*)^. The duty ratio is defined as *r*_*duty*_ = *t*_*pulse-width*_ / *t*_*period*_, which is an important parameter in determining the thermal response of the skin.

It is universally accepted that the denaturation of protein can be used to measure the degree of thermal damage. The denaturation rate defined by^21^ is given as:

11 $$ K\left( T \right) = A\exp \left( { - \frac{{E_{a} }}{RT}} \right) $$

where, *A* represents the frequency factor, *R* for the universal gas constant and *E*_α_ for the activation energy of the denaturation reaction. The thermal damage is determined by temperature and exposure duration, which can be evaluated as^21^:

12 $$ \Omega \left( {T,t} \right) = \int_{0}^{t} {A\exp \left( { - \frac{{E_{\alpha } }}{RT}} \right)dt} $$

## Exact solution for governing equations

### Solution in terms of Green’s function

In 1985, Frankel^35^ presented the Green’s function approach for hyperbolic heat conduction in a one-dimensional medium. In the following, we will extend this procedure into DPL model in a three-dimensional finite medium and develop the general solution to Eqs. (3)–(10). The results will be used to solve the present heat conduction problem.

Denote an operator *L* as following:

13 $$ L = \tau_{q} \frac{{\partial^{2} }}{{\partial t^{2} }} + \left( {\tau_{q} \overline{w}_{b} + 1} \right)\frac{\partial }{\partial t} - \alpha \nabla^{2} - a\tau_{T} \frac{\partial }{\partial t}\nabla^{2} + \overline{w}_{b} $$

where, $$\overline{w}_{b} = \frac{{w_{b} \rho_{b} C_{b} }}{\rho C}$$, $$\nabla^{2} = \frac{{\partial^{2} }}{{\partial x^{2} }} + \frac{{\partial^{2} }}{{\partial y^{2} }} + \frac{{\partial^{2} }}{{\partial z^{2} }}$$.

Then Eq. (3) can be expressed as:

14 $$ L\theta = q\left( {x,y,z,t} \right) $$

where,

15 $$ q\left( {x,y,z,t} \right) = \frac{1}{\rho C}\left[ {Q_{m} + Q_{l} + \tau_{q} \left( {\frac{{\partial Q_{l} }}{\partial t} + \frac{{\partial Q_{m} }}{\partial t}} \right)} \right] $$

According to the procedure presented by^35^, the following integration is defined:

16 $$ I = \int_{0}^{t + \varepsilon } {\int_{0}^{h} {\int_{0}^{b} {\int_{0}^{l} {G\left( {\left. {\vec{r},t} \right|\vec{r}^{\prime},\tau } \right)L^{\prime}\left[ {\theta \left( {\vec{r}^{\prime},\tau } \right)} \right]dx^{\prime}dy^{\prime}dz^{\prime}d\tau } } } } $$

where $G\left( {\left. {\vec{r},t} \right|\vec{r}^{\prime},\tau } \right)$ is the corresponding Green’s function for Eq. (3), which is written to represent the “effect/cause” relationship. Here we denote $\vec{r} = \left( {x,y,z} \right)$ and $\vec{r}^{\prime} = \left( {x^{\prime},y^{\prime},z^{\prime}} \right)$ to show the point. The introduction of *ε* > 0 is to invoke causality at a later time in the analysis^35^. The operator *L’* in Eq. (14) with respect to the cause variables *x’*, *y’*, *z’* and *τ* can be written as:

17 $$ L^{\prime} = \tau_{q} \frac{{\partial^{2} }}{{\partial \tau^{2} }} + \left( {1 + \tau_{q} \overline{w}_{b} } \right)\frac{\partial }{\partial \tau } - \alpha \nabla^{{\prime}{2}} - \tau_{T} \alpha \frac{\partial }{\partial \tau }\nabla^{{\prime}{2}} + \overline{w}_{b} $$

where, $$\nabla^{{\prime}{2}} = \frac{{\partial^{2} }}{{\partial x^{{\prime}{2}} }} + \frac{{\partial^{2} }}{{\partial y^{{\prime}{2}} }} + \frac{{\partial^{2} }}{{\partial z^{{\prime}{2}} }}$$.

Integration of the right part of Eq. (16) by parts yields the following expression:

18 $$ I = \mathop {\lim }\limits_{\varepsilon \to 0} \left\{ {I_{1} + I_{2} + I_{3} + I_{4} + I_{5} } \right\} $$

where,

19 $$ I_{1} = \int_{0}^{h} {\int_{0}^{b} {\int_{0}^{l} {\left[ \begin{gathered} \left. {\left( {1 + \tau_{q} \overline{w}_{b} } \right)G\theta } \right|_{\tau = 0}^{\tau = t + \varepsilon } + \left. {\tau_{q} G\frac{\partial \theta }{{\partial \tau }}} \right|_{\tau = 0}^{\tau = t + \varepsilon } - \left. {\tau_{q} \theta \frac{\partial G}{{\partial \tau }}} \right|_{\tau = 0}^{\tau = t + \varepsilon } \hfill \\ - \left. {\tau_{T} \alpha G\frac{{\partial^{2} \theta }}{{\partial x^{{\prime}{2}} }}} \right|_{\tau = 0}^{\tau = t + \varepsilon } - \left. {\tau_{T} \alpha G\frac{{\partial^{2} \theta }}{{\partial y^{{\prime}{2}} }}} \right|_{\tau = 0}^{\tau = t + \varepsilon } - \left. {\tau_{T} \alpha G\frac{{\partial^{2} \theta }}{{\partial z^{{\prime}{2}} }}} \right|_{\tau = 0}^{\tau = t + \varepsilon } \hfill \\ \end{gathered} \right]} } } dx^{\prime}dy^{\prime}dz^{\prime} $$

20 $$ I_{2} = \int_{0}^{t + \varepsilon } {\int_{0}^{h} {\int_{0}^{b} {\left[ \begin{gathered} \left. { - \alpha G\frac{\partial \theta }{{\partial x^{\prime}}}} \right|_{{x^{\prime} = 0}}^{l} + \left. {\alpha \frac{\partial G}{{\partial x^{\prime}}}\theta } \right|_{{x^{\prime} = 0}}^{l} \hfill \\ + \left. {\tau_{T} \alpha \frac{\partial G}{{\partial \tau }}\frac{\partial \theta }{{\partial x^{\prime}}}} \right|_{{x^{\prime} = 0}}^{l} - \left. {\tau_{T} \alpha \frac{{\partial^{2} G}}{{\partial \tau \partial x^{\prime}}}\theta } \right|_{{x^{\prime} = 0}}^{l} \hfill \\ \end{gathered} \right]} } } dy^{\prime}dz^{\prime}d\tau $$

21 $$ I_{3} = \int_{0}^{t + \varepsilon } {\int_{0}^{h} {\int_{0}^{l} {\left[ \begin{gathered} \left. { - \alpha G\frac{\partial \theta }{{\partial y^{\prime}}}} \right|_{{y^{\prime} = 0}}^{b} + \left. {\alpha \frac{\partial G}{{\partial y^{\prime}}}\theta } \right|_{{y^{\prime} = 0}}^{b} \hfill \\ + \left. {\tau_{T} \alpha \frac{\partial G}{{\partial \tau }}\frac{\partial \theta }{{\partial y^{\prime}}}} \right|_{{y^{\prime} = 0}}^{b} - \left. {\tau_{T} \alpha \frac{{\partial^{2} G}}{{\partial \tau \partial y^{\prime}}}\theta } \right|_{{y^{\prime} = 0}}^{b} \hfill \\ \end{gathered} \right]} } } dx^{\prime}dz^{\prime}d\tau $$

22 $$ I_{4} = \int_{0}^{t + \varepsilon } {\int_{0}^{b} {\int_{0}^{l} {\left[ \begin{gathered} \left. { - \alpha G\frac{\partial \theta }{{\partial z^{\prime}}}} \right|_{{z^{\prime} = 0}}^{h} + \left. {\alpha \frac{\partial G}{{\partial z^{\prime}}}\theta } \right|_{{z^{\prime} = 0}}^{h} \hfill \\ + \left. {\tau_{T} \alpha \frac{\partial G}{{\partial \tau }}\frac{\partial \theta }{{\partial z^{\prime}}}} \right|_{{z^{\prime} = 0}}^{h} - \left. {\tau_{T} \alpha \frac{{\partial^{2} G}}{{\partial \tau \partial z^{\prime}}}\theta } \right|_{{z^{\prime} = 0}}^{h} \hfill \\ \end{gathered} \right]dx^{\prime}dy^{\prime}d\tau } } } $$

23 $$ I_{5} = \int_{0}^{t + \varepsilon } {\int_{0}^{h} {\int_{0}^{b} {\int_{0}^{l} {\theta \left( {\vec{r}^{\prime},\tau } \right)L^{\prime\;*} \left[ {G\left( {\left. {\vec{r},t} \right|\vec{r}^{\prime},\tau } \right)} \right]dx^{\prime}dy^{\prime}dz^{\prime}d\tau } } } } $$

where L’* is the formal adjoint operator of the operator *L’*, which can be written as^35^:

24 $$ L^{\prime\;*} = \tau_{q} \frac{{\partial^{2} }}{{\partial \tau^{2} }} - \left( {1 + \tau_{q} \overline{w}_{b} } \right)\frac{\partial }{\partial \tau } - \alpha \nabla^{{\prime}{2}} + \tau_{T} \alpha \frac{\partial }{\partial \tau }\left( {\nabla^{{\prime}{2}} } \right) + \overline{w}_{b} $$

The Green’s function for Eq. (3) is chosen to satisfy the following auxiliary problem:

25 $$ L^{\prime\;*} \left[ {G\left( {\left. {\vec{r},t} \right|\vec{r}^{\prime},\tau } \right)} \right] = \delta \left( {x - x^{\prime}} \right)\delta \left( {y - y^{\prime}} \right)\delta \left( {z - z^{\prime}} \right)\delta \left( {t - \tau } \right) $$

And the following homogeneous boundary conditions and additional requirement should be satisfied:

26 $$ \left\{ \begin{gathered} \left. G \right|_{{x^{\prime} = 0}} = \left. G \right|_{{x^{\prime} = l}} = 0 \hfill \\ \left. G \right|_{{y^{\prime} = 0}} = \left. G \right|_{{y^{\prime} = b}} = 0 \hfill \\ \left. G \right|_{{z^{\prime} = h}} \hfill \\ \left. {\left( { - k\frac{\partial G}{{\partial z^{\prime}}} + h_{1} G} \right)} \right|_{{z^{\prime} = 0}} = 0 \hfill \\ \end{gathered} \right. $$

27 $$ \left\{ \begin{gathered} G\left( {\left. {\vec{r},t} \right|\vec{r}^{\prime},\tau } \right) = 0,\;\;t < \tau \hfill \\ \frac{{\partial G\left( {\left. {\vec{r},t} \right|\vec{r}^{\prime},\tau } \right)}}{\partial \tau } = 0,\;\;t < \tau \hfill \\ \end{gathered} \right. $$

Equation (27) represents the causality principle, which is merely a statement that no effect can be experienced prior to a cause . Substituting Eqs. (19)–(23) into Eq. (18) and taking the limit as $\varepsilon \to 0$ in Eqs. (19)–(23) yields

28 $$ I_{5} = \theta \left( {\vec{r},t} \right) $$

So the temperature distribution of the skin model in the present study can be expressed with the usage of Green’s function as following:

29 $$ \begin{gathered} \theta \left( {\vec{r},t} \right) = \int_{0}^{t} {\int_{0}^{h} {\int_{0}^{b} {\int_{0}^{l} {G\left( {\left. {\vec{r},t} \right|\vec{r}^{\prime},\tau } \right)q\left( {\vec{r}^{\prime},\tau } \right)dx^{\prime}dy^{\prime}dz^{\prime}d\tau } } } } \hfill \\ \;\;\;\;\;\;\;\;\;\;\;\; - I_{1} - I_{2} - I_{3} - I_{4} \hfill \\ \end{gathered} $$

### Determination of the Green’s function

The Green’s function can be derived from Eqs. (25)–(27). The associated equation can be written as:

30 $$ \begin{gathered} \tau_{q} \frac{{\partial^{2} G}}{{\partial \tau^{2} }} - \left( {1 + \tau_{q} \overline{w}_{b} } \right)\frac{\partial G}{{\partial \tau }} - \alpha \nabla^{{\prime}{2}} G + \tau_{T} \alpha \frac{\partial }{\partial \tau }\left( {\nabla^{{\prime}{2}} G} \right) + \overline{w}_{b} G \hfill \\ = \delta \left( {x - x^{\prime}} \right)\delta \left( {y - y^{\prime}} \right)\delta \left( {z - z^{\prime}} \right)\delta \left( {t - \tau } \right) \hfill \\ \end{gathered} $$

The mode functions satisfying the boundary conditions are^36^:

31 $$ \left\{ \begin{gathered} X_{m} \left( {\eta_{m} ,x} \right) = \sin \left( {\eta_{m} x} \right) \hfill \\ Y_{n} \left( {\gamma_{n} ,y} \right) = \sin \left( {\gamma_{n} y} \right) \hfill \\ Z_{s} \left( {\zeta_{s} ,z} \right) = C_{s} \cos \left( {\zeta_{s} z} \right) + \sin \left( {\zeta_{s} z} \right) \hfill \\ \end{gathered} \right. $$

where, $\eta_{m} = \frac{m\pi }{l}$, $\gamma_{n} = \frac{n\pi }{b}$, $C_{s} = - \frac{{\sin \left( {\zeta_{s} h} \right)}}{{\cos \left( {\zeta_{s} h} \right)}}$. According to the boundary conditions, ζ_*s*_ can be derived by the following equation:

32 $$ h_{1} \sin \left( {\zeta_{s} h} \right) + k\zeta_{s} \cos \left( {\zeta_{s} h} \right) = 0 $$

By operating on Eq. (30) with $\int_{0}^{h} {\int_{0}^{b} {\int_{0}^{l} {X_{m} \left( {\eta_{m} ,x^{\prime}} \right)Y_{n} \left( {\gamma_{n} ,y^{\prime}} \right)Z_{s} \left( {\zeta_{s} ,z^{\prime}} \right)dx^{\prime}dy^{\prime}dz^{\prime}} } }$ , and incorporating the homogeneous boundary conditions of Eq. (26), one can obtain:

33 $$ \begin{gathered} \tau_{q} \frac{{\partial^{2} \overline{G}_{mns} }}{{\partial \tau^{2} }} - \left( {1 + \tau_{q} \overline{w}_{b} + \tau_{T} \lambda_{mns}^{2} } \right)\frac{{\partial \overline{G}_{mns} }}{\partial \tau } + \left( {\overline{w}_{b} + \lambda_{mns}^{2} } \right)\overline{G}_{mns} \hfill \\ = X_{m} \left( {\eta_{m} ,x} \right)Y_{n} \left( {\gamma_{n} ,y} \right)Z_{s} \left( {\zeta_{s} ,z} \right)\delta \left( {t - \tau } \right) \hfill \\ \end{gathered} $$

where,

34 $$ \lambda_{mns}^{2} = \alpha \left( {\eta_{m}^{2} + \gamma_{n}^{2} + \zeta_{s}^{2} } \right) $$

35 $$ \overline{G}_{mns} \left( \tau \right) = \int_{0}^{h} {\int_{0}^{b} {\int_{0}^{l} {G\left( {\left. {\vec{r},t} \right|\vec{r}^{\prime},\tau } \right)X_{m} \left( {\eta_{m} ,x^{\prime}} \right)Y_{n} \left( {\gamma_{n} ,y^{\prime}} \right)Z_{s} \left( {\zeta_{s} ,z^{\prime}} \right)dx^{\prime}dy^{\prime}dz^{\prime}} } } $$

The inversion formula is

36 $$ G\left( {\left. {\vec{r},t} \right|\vec{r}^{\prime},\tau } \right) = \sum\limits_{m = 1}^{\infty } {\sum\limits_{n = 1}^{\infty } {\sum\limits_{s = 1}^{\infty } {\frac{{X_{m} \left( {\eta_{m} ,x^{\prime}} \right)Y_{n} \left( {\gamma_{n} ,y^{\prime}} \right)Z_{s} \left( {\zeta_{s} ,z^{\prime}} \right)}}{{M_{m} N_{n} S_{s} }}\overline{G}_{mns} \left( \tau \right)} } } $$

where $$M_{m} = \int_{0}^{l} {X_{m}^{2} \left( {\eta_{m} ,x^{\prime}} \right)dx^{\prime}}$$, $$N_{n} = \int_{0}^{b} {Y_{n}^{2} \left( {\gamma_{n} ,y^{\prime}} \right)dy^{\prime}}$$, $$S_{s} = \int_{0}^{h} {Z_{s}^{2} \left( {\zeta_{s} ,z^{\prime}} \right)dz^{\prime}}$$.

With Eq. (27) under consideration, the solution for Eq. (33) can be written as:

37 $$ \overline{G}_{mns} \left( \tau \right) = \frac{{X_{m} \left( {\eta_{m} ,x} \right)Y_{n} \left( {\gamma_{n} ,y} \right)Z_{s} \left( {\zeta_{s} ,z} \right)}}{{\tau_{q} \beta_{2} }}\exp \left[ { - \beta_{1} \left( {t - \tau } \right)} \right]\sinh \left[ {\beta_{2} \left( {t - \tau } \right)} \right] $$

where, $$\beta_{1} = \frac{{1 + \tau_{q} \overline{w}_{b} + \tau_{T} \lambda_{mns}^{2} }}{{2\tau_{q} }}$$, $$\beta_{2} = \frac{{\sqrt {\left( {1 + \tau_{q} \overline{w}_{b} + \tau_{T} \lambda_{mns}^{2} } \right)^{2} - 4\tau_{q} \left( {\overline{w}_{b} + \lambda_{mns}^{2} } \right)} }}{{2\tau_{q} }}$$.

Substitution of Eq. (37) into Eq. (36) yields the Green’s function for the governing equations as:

38 $$ G\left( {\left. {\vec{r},t} \right|\vec{r}^{\prime},\tau } \right) = \sum\limits_{m = 1}^{\infty } {\sum\limits_{n = 1}^{\infty } {\sum\limits_{s = 1}^{\infty } {\left\{ \begin{gathered} \frac{{X_{m} \left( {\eta_{m} ,x} \right)Y_{n} \left( {\gamma_{n} ,y} \right)Z_{s} \left( {\zeta_{s} ,z} \right)}}{{M_{m} N_{n} S_{s} }}\exp \left[ { - \beta_{1} \left( {t - \tau } \right)} \right] \hfill \\ \cdot \frac{{X_{m} \left( {\eta_{m} ,x^{\prime}} \right)Y_{n} \left( {\gamma_{n} ,y^{\prime}} \right)Z_{s} \left( {\zeta_{s} ,z^{\prime}} \right)}}{{\tau_{q} \beta_{2} }}\sinh \left[ {\beta_{2} \left( {t - \tau } \right)} \right] \hfill \\ \end{gathered} \right\}} } } $$

### Solution for the nonhomogeneous equation

With the boundary conditions and the initial conditions under consideration, the solution for Eq. (3) can be expressed as:

39 $$ \begin{gathered} \theta \left( {\vec{r},t} \right) = \int_{0}^{t} {\int_{0}^{h} {\int_{0}^{b} {\int_{0}^{l} {G\left( {\left. {\vec{r},t} \right|\vec{r}^{\prime},\tau } \right)q\left( {\vec{r}^{\prime},\tau } \right)dx^{\prime}dy^{\prime}dz^{\prime}d\tau } } } } \\ \quad + \int_{0}^{t} {\int_{0}^{b} {\int_{0}^{l} {\left( { - \alpha \frac{{f_{1} }}{k}\left. G \right|_{{z^{\prime} = 0}} + \alpha \tau_{T} \frac{{f_{1} }}{k}\left. {\frac{\partial G}{{\partial \tau }}} \right|_{{z^{\prime} = 0}} } \right)} } } dx^{\prime}dy^{\prime}d\tau \\ \end{gathered} $$

Substitute Eqs. (4)–(8), (15) and (38) into Eq. (39), and the solution for the nonhomogeneous Eq. (3) can be derived as:

40 $$ \theta \left( {\vec{r},t} \right) = \sum\limits_{m = 1}^{\infty } {\sum\limits_{n = 1}^{\infty } {\sum\limits_{s = 1}^{\infty } {\frac{{X_{m} \left( {\eta_{m} ,x} \right)Y_{n} \left( {\gamma_{n} ,y} \right)Z_{s} \left( {\zeta_{s} ,z} \right)}}{{M_{m} N_{n} S_{s} \tau_{q} \beta_{2} }}\left\{ \begin{gathered} \frac{{G_{m2} G_{n2} G_{s2} \left( {P_{2} + \tau_{q} \psi_{2} } \right)}}{\rho C} \hfill \\ - \frac{{\alpha f_{1} }}{k}Z_{s} \left( {\zeta_{s} ,0} \right)G_{m1} G_{n1} P_{1} \hfill \\ + \frac{{q_{m} G_{m1} G_{n1} G_{s1} P_{1} }}{\rho C} \hfill \\ + \frac{{\alpha \tau_{T} f_{1} }}{k}Z_{s} \left( {\zeta_{s} ,0} \right)G_{m1} G_{n1} \psi_{1} \hfill \\ \end{gathered} \right\}} } } $$

where,

$$ \begin{aligned}    & G_{{n1}}  = \int_{0}^{b} {Y_{n} \left( {\gamma _{n} ,y} \right)dy} ,\quad G_{{s1}}  = \int_{0}^{h} {Z_{s} \left( {\zeta _{s} ,z} \right)dz} ,\quad G_{{m2}}  = \int_{0}^{l} {X_{m} \left( {\eta _{m} ,x} \right)Q_{1} \left( x \right)dx} , \\     & G_{{n2}}  = \int_{0}^{b} {Y_{n} \left( {\gamma _{n} ,y} \right)Q_{2} \left( y \right)dy} ,\quad G_{{s2}}  = \int_{0}^{h} {Z_{s} \left( {\zeta _{s} ,z} \right)\varphi \left( z \right)dz} , \\     & P_{1}  = \int_{0}^{t} {\exp \left[ { - \beta _{1} \left( {t - \tau } \right)} \right]sh\left[ {\beta _{2} \left( {t - \tau } \right)} \right]d\tau } , \\     & P_{2}  = \int_{0}^{t} {\exp \left[ { - \beta _{1} \left( {t - \tau } \right)} \right]sh\left[ {\beta _{2} \left( {t - \tau } \right)} \right]Q_{3} \left( \tau  \right)d\tau } , \\     & \psi _{1}  =  - \exp \left( { - \beta _{1} t} \right)\sinh\left( {\beta _{2} t} \right), \\     & \psi _{2}  = \int_{0}^{t} {\exp \left[ { - \beta _{1} \left( {t - \tau } \right)} \right]\sinh\left[ {\beta _{2} \left( {t - \tau } \right)} \right]\frac{{\partial Q_{3} \left( \tau  \right)}}{{\partial \tau }}d\tau }  \\  \end{aligned}  $$

## Numerical results and discussions

The parameters of the heat source and the skin in the present study are shown in Tables 1 and 2, respectively^19,37,38^. The core temperature of human body is usually set as 37 °C, and the temperature of arterial blood and the initial temperature of the vivo skin can also be set as *T*_*a*_ = 37 °C and *T*_*0*_ = 37 °C. The ambient temperature is described as *T*_*e*_ = 20 °C .

**Table 1:** Parameters of laser beam.

Parameters	Values
*I*_*0*_, parameter of laser power(W/m^3^)	3 × 10^4^
*R*_*a*_, energy reflectivity coefficient	0.024
*R*_*0*_, size parameter of laser spot (m)	2 × 10^–3^

**Table 2:** Thermal parameters of the skin.

Parameters	Values
Skin specific heat (J/kg∙K)	2348
Skin density (kg/m^3^)	911
Blood specific heat (J/kg∙K)	3770
Blood density (kg/m^3^)	1060
Thermal conductivity (W/m∙K)	0.235
Metabolic heat Generation (W/m^3^)	368.1
Length (m)	0.02
Width (m)	0.02
Thickness (m)	0.01
Blood perfusion (s^−1^)	0.0016

The burn assessment model employed proposed by^21^ is used, which is based on the description of protein thermal denaturation rate. And the parameters of the model are shown in Table 3^19^. It is accepted that: $0.53 \le \Omega < 1$ represents the first-degre burn, $1 \le \Omega < 10^{4}$ for the second degree-burn (irreversible burn) and $\Omega \ge 10^{4}$ for the third-degree burn^15,39^.

**Table 3:** Parameters for thermal damage prediction.

Temperature range (^o^C)	*E*_*α*_/*R* (K)	*A*, frequency factor (s^−1^)
*T* ≤ 55	7.5 × 10^4^	3.1 × 10^98^
*T* > 55	3.54 × 10^4^	5.0 × 10^45^

The exact values of the two phase lags for the skin are still under study. However, it is widely agreed that the values fall to the order of magnitude ~ s^40–42^. So the phase lags will be chosen based on this range.

### Degeneration to the Fourier’s law

The DPL heat transfer model can degenerate to the Pennes model by setting *τ*_*q*_ = *τ*_*T*_ = 0. The expression for the distribution of the temperature rise based on the Pennes model can be expressed as:

41 $$ \begin{gathered} \theta^{*} \left( {\vec{r},t} \right) = \int_{0}^{t} {\int_{0}^{h} {\int_{0}^{b} {\int_{0}^{l} {G^{*} \left( {\left. {\vec{r},t} \right|\vec{r}^{\prime},\tau } \right)q\left( {\vec{r}^{\prime},\tau } \right)dx^{\prime}dy^{\prime}dz^{\prime}d\tau } } } } \\ \; - \int_{0}^{t} {\int_{0}^{b} {\int_{0}^{l} {\alpha \frac{{f_{1} }}{k}\left. {G^{*} } \right|_{{z^{\prime} = 0}} } } } dx^{\prime}dy^{\prime}d\tau \\ \end{gathered} $$

where, the $G^{*} \left( {\left. {\vec{r},t} \right|\vec{r}^{\prime},\tau } \right)$ is the Green’s function for the Pennes model, which can be written as:

42 $$ G^{*} \left( {\left. {\vec{r},t} \right|\vec{r}^{\prime},\tau } \right) = \sum\limits_{m = 1}^{\infty } {\sum\limits_{n = 1}^{\infty } {\sum\limits_{s = 1}^{\infty } {\left\{ \begin{gathered} \frac{{X_{m} \left( {\eta_{m} ,x} \right)Y_{n} \left( {\gamma_{n} ,y} \right)Z_{s} \left( {\zeta_{s} ,z} \right)}}{{M_{m} N_{n} S_{s} }} \hfill \\ \cdot X_{m} \left( {\eta_{m} ,x^{\prime}} \right)Y_{n} \left( {\gamma_{n} ,y^{\prime}} \right)Z_{s} \left( {\zeta_{s} ,z^{\prime}} \right) \hfill \\ \cdot \exp \left[ { - \left( {\lambda^{2} + \overline{w}_{b} } \right)\left( {t - \tau } \right)} \right] \hfill \\ \end{gathered} \right\}} } } $$

Substituting Eq. (42) into Eq. (41) can yield the following formulas:

43 $$ \theta^{*} \left( {\vec{r},t} \right) = \sum\limits_{m = 1}^{\infty } {\sum\limits_{n = 1}^{\infty } {\sum\limits_{s = 1}^{\infty } {\frac{{X_{m} \left( {\eta_{m} ,x} \right)Y_{n} \left( {\gamma_{n} ,y} \right)Z_{s} \left( {\zeta_{s} ,z} \right)}}{{M_{m} N_{n} S_{s} }}\left\{ \begin{gathered} \frac{{G_{m2} G_{n2} G_{s2} P_{2}^{*} }}{\rho C} - \frac{{\alpha f_{1} }}{k}Z_{s} \left( {\zeta_{s} ,0} \right)G_{m1} G_{n1} P_{1}^{*} \hfill \\ + \frac{{q_{m} G_{m1} G_{n1} G_{s1} P_{1}^{*} }}{\rho C} \hfill \\ \end{gathered} \right\}} } } $$

where,

44 $$ P_{1}^{*} = \int_{0}^{t} {\exp \left[ { - \left( {\lambda_{mns}^{2} + \overline{w}_{b} } \right)\left( {t - \tau } \right)} \right]d\tau } $$

45 $$ P_{2}^{*} = \int_{0}^{t} {\exp \left[ { - \left( {\lambda_{mns}^{2} + \overline{w}_{b} } \right)\left( {t - \tau } \right)} \right]Q_{3} \left( \tau \right)d\tau } $$

**Figure 4:**
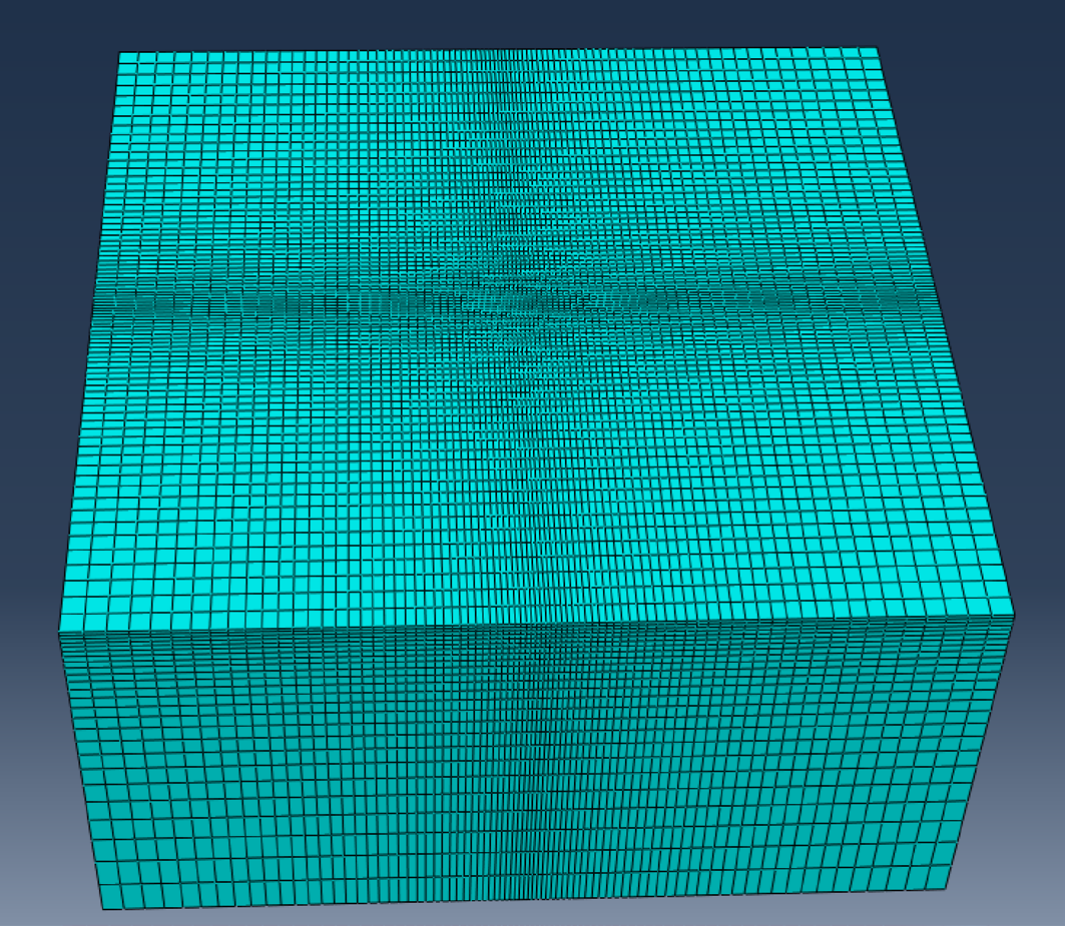
The FEM model of the skin (created by Abaqus 6.14, https://www.3ds.com/zh/products-services/simulia/products/abaqus/).

A finite element simulation is carried out to validate the analytical solution derived in the present study (by ABAQUS-6.14, Dassault Systemes Simulia Corp.). For ease of comparing the numerical and analytical solutions, the value of blood perfusion (*w*_*b*_) is set to be zero and the analytical solution is reduced to the Pennes model. The Abaqus software is employed for the finite element analysis. The skin model is meshed into 160,000 elements, which are set to be 8-node linear heat transfer bricks. The grids is densified at the center point of the top surface, as is shown in Fig. 4. With the usage of Fortran, the multi-pulse heat source is applied as a body heat generation with the expression of the heat source given by Eqs. (4)–(8). The simulation is set to be transient and the time period (20 s) is divided into two stages: the heating stage (0 ~ 3 s) and the non-heating stage (3 ~ 20 s). The temperature increment of the two stages are 0.01 s and 0.1 s, respectively.

**Figure 5:**
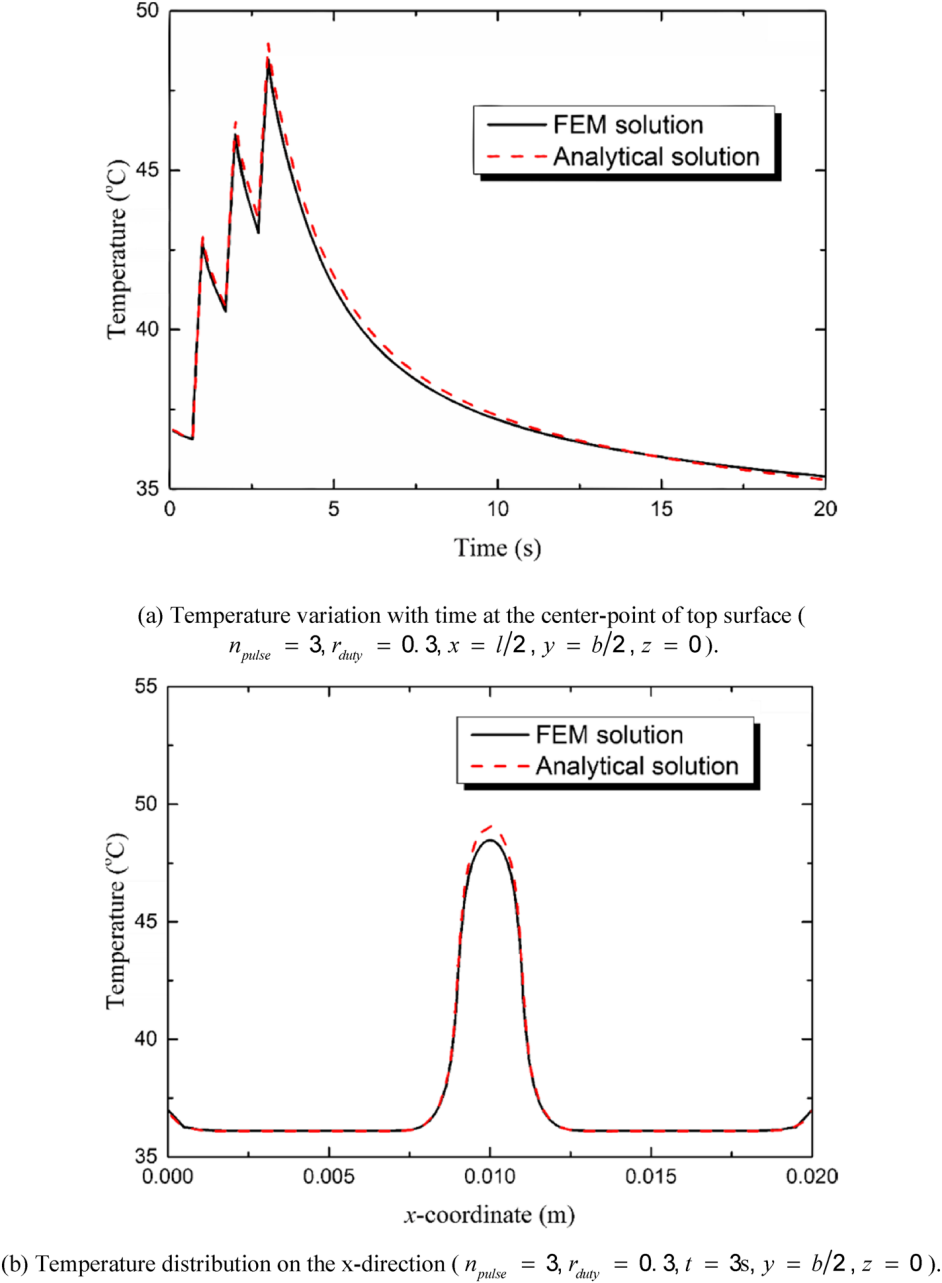
Comparison between FEM and analytical solutions.

Figure 5 shows the comparison of the results derived by the FEM and analytical solution. The heat source involves three pulses (*n*_*pulse*_ = 3) and the duty ratio is set to be 0.3 (*r*_*duty*_ = 0.3, *t*_*period*_ = 1 s and *t*_*pulse-width*_ = 0.3 s). The boundary condition on the top surface of the model is natural convection condition. And the temperature on the other surfaces is equal to the core temperature *T*_*0*_. The value of the convective heat transfer coefficient is set to be *h*_*1*_ = 10 W/(m^2^ K) with the assumption of natural convection between the skin and the air. It is shown in Fig. 4 that the results derived by FEM and analytical solution are in good agreement.

### Results based on DPL model

In the following, the thermal response of the skin irradiated by repetitive laser pulses are investigated based on the DPL model.

**Figure 6:**
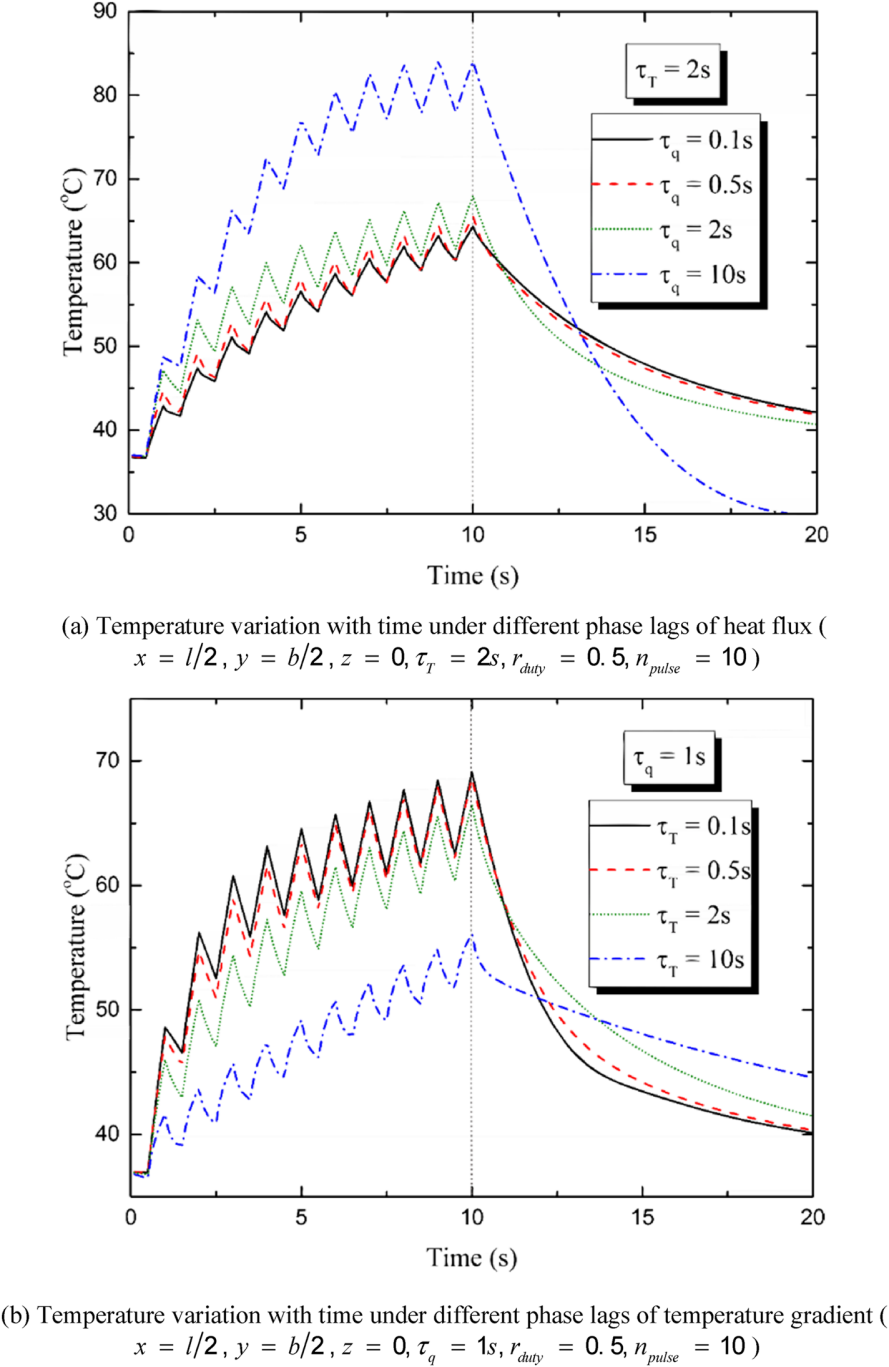

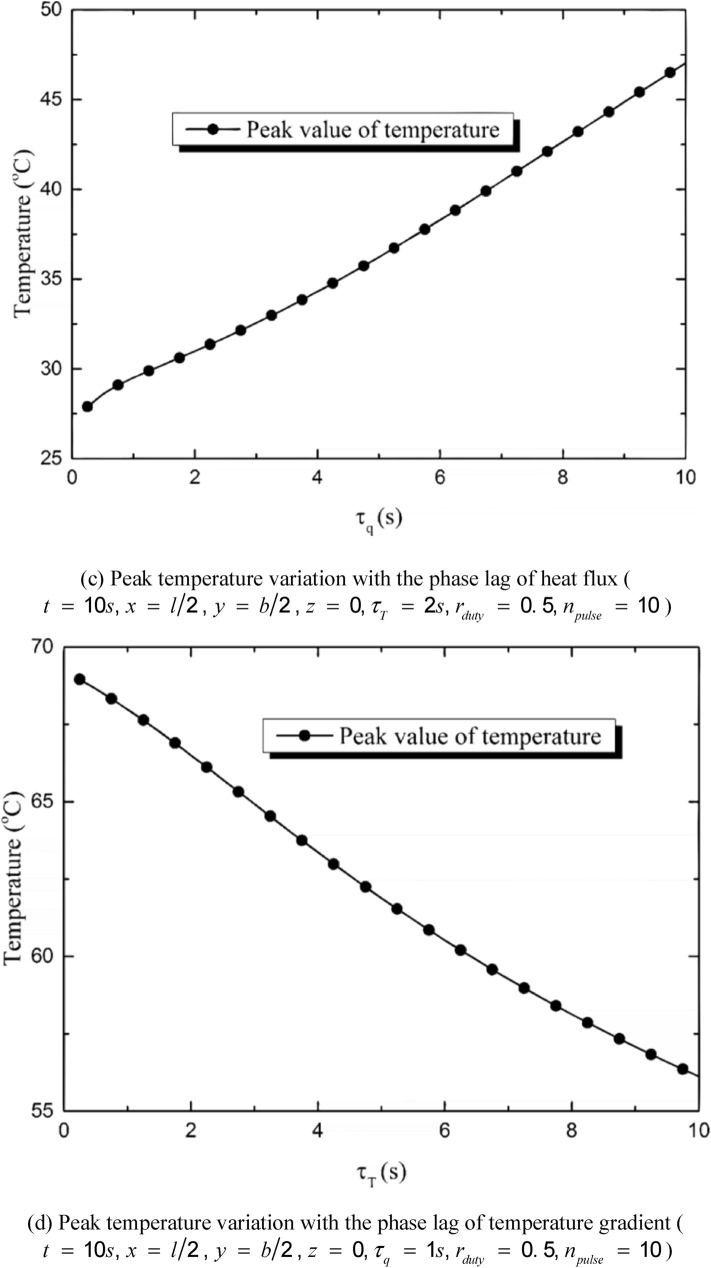
Influences of the phase lags on the temperature variation induced by repetitive laser pulses.

Figure 6 shows the influences of the heat flux phase lag *τ*_*q*_ and temperature gradient phase lag *τ*_*T*_ on the temperature distribution induced by repetitive laser pulses. The pulse number is set to be *n*_*pulse*_ = 10 and the duty ratio is *r*_*duty*_ = 0.5 (*t*_*period*_ = 1 s, *t*_*pulse-width*_ = 0.5 s). It is shown in Fig. 6 that the temperature variation can be divided into two stages: the heating stage and the non-heating stage. The heating stage is the first ten seconds during which the laser pulses irradiate on the skin and the temperature rises in a serrated form^43,44^. The non-heating stage is the latter ten seconds when the heat source is taken off and the temperature decreases due to the heat transfer and the convective heat dissipation. Figure 6 (a) and (b) show the temperature variation with time at the center point of the irradiated region (*x* = *l*/2, *y* = *b*/2, *z* = 0). A great *τ*_*q*_ leads to a tardy heat conduction progress, which means the temperature variation shows up before the heat transfer starts. On the opposite, a greater *τ*_*T*_ leads to a faster heat balance progress, which means that more heat flux will be transferred from high-temperature region to the low-temperature region before the temperature variation appears. It is shown in Fig. 6a that when *τ*_*q*_ takes a great value, the temperature rises rapidly during the first three pulse periods and then the growth tendency slows down. During the last three pulse periods, the tendency of temperature growth nearly disappears especially when *τ*_*q*_ = 10 s. In the non-heating stage, a greater *τ*_*q*_ leads to a faster cooling rate because of the fluctuation characteristic caused by *τ*_*q*_. If *τ*_*q*_ is great enough, the temperature can be even lower than the initial temperature. Figure 6b demonstrates that the temperature rise induced by every laser pulse tends to be the same and the tendency of temperature growth tends to be linear when *τ*_*T*_ increasing from 0.1 s to 10 s. The larger the value of *τ*_*T*_ is, the more smoothly the temperature decreases during the non-heating stage. Figure 6c,d show the peak temperature variations (appearing at the moment *t* = 10 s) on *τ*_*q*_ and *τ*_*T*_. It can be found that the peak temperature of the skin induced by the multi-pulse heat source increases with the increment of *τ*_*q*_ and the decrement of *τ*_*T*_. The phase lag of heat flux strengthens the influence of external heat source and leads to intense temperature variation by hindering the heat transfer process. On the contrary, the phase lag of the temperature leads to gentle temperature change by promoting the thermal balance process.

**Figure 7:**
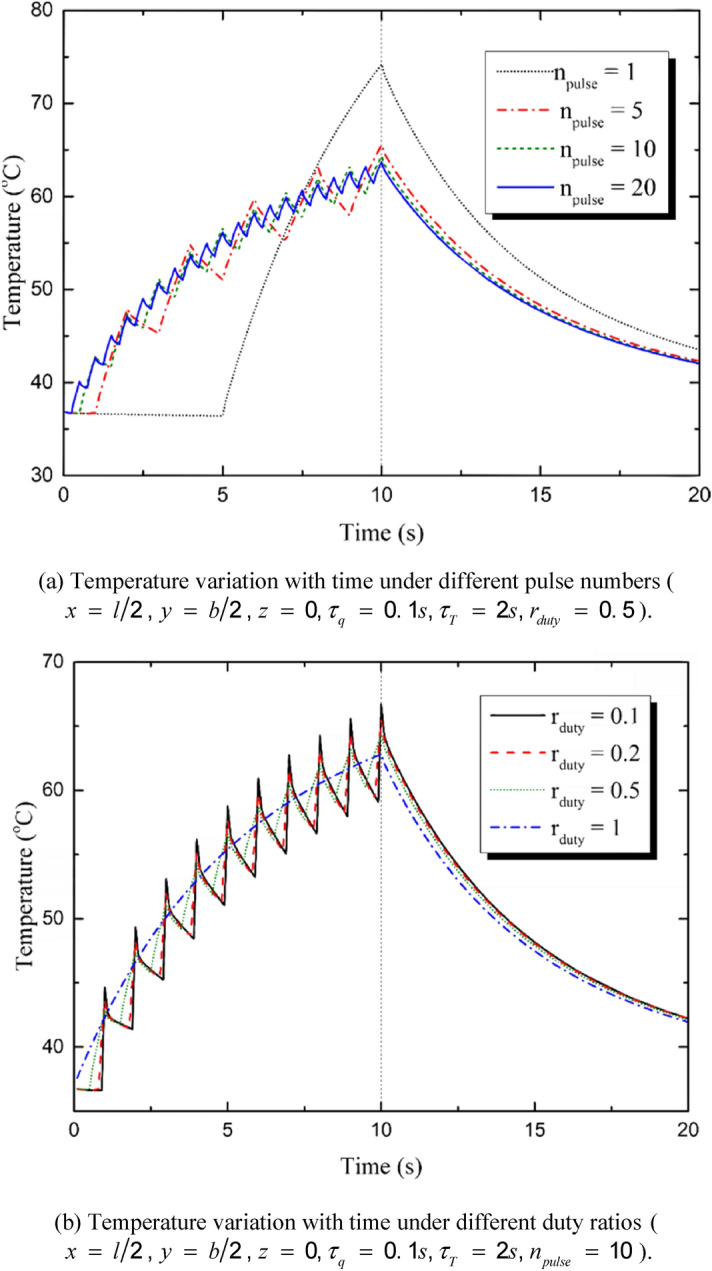

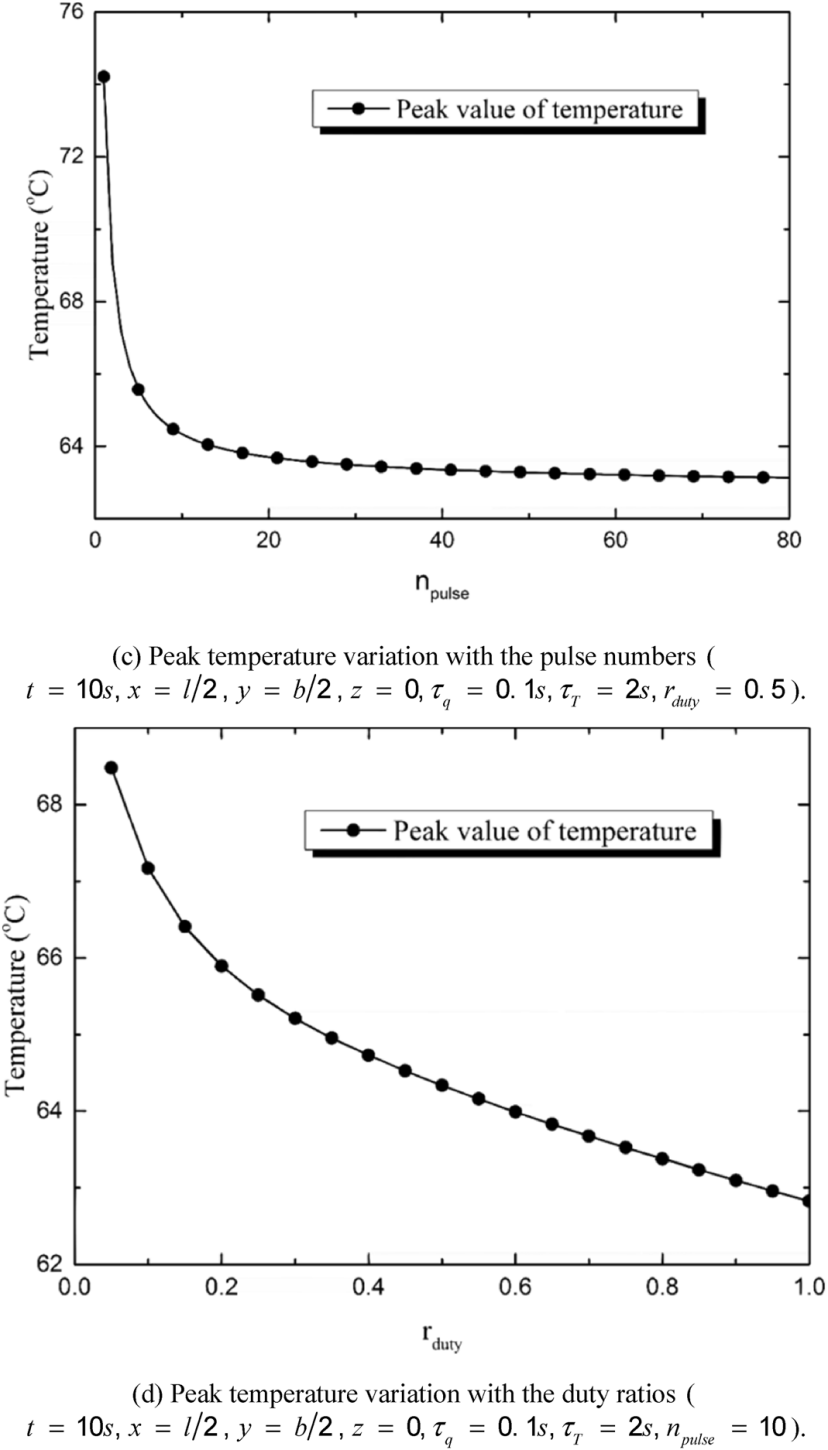
Influences of the laser parameters on the temperature variation.

Figure 7a shows the influences of the pulse number on the temperature variation of the skin. In the present study, the total input energy is assumed to be constant and not change with the pulse number. It is shown in Fig. 7a that the amplitude of temperature rise is the maximum when *n*_*pulse*_ = 1 and it decreases obviously with the increment of *n*_*pulse*_. Figure 7b shows the influences of duty ratio on the temperature variation. The vibration amplitude of temperature increases with the decrement of duty ratio. However, the overall level of the temperature changes little. When *r*_*duty*_ = 1, the multi-pulse heat source regenerate to a constant heat source and the temperature changes smoothly. Figure 7c shows the peak temperature variation with the pulse number. The temperature reaches the maximum value when *n*_*pulse*_ = 1 and then reduces rapidly. With the increasing of *n*_*pulse*_, the reduction tendency keeps attenuating and the value of the peak temperature tends to be stable. Figure 7d shows the peak temperature variation with the duty ratio. The increment of the duty ratio leads to the decrement of the peak temperature by decreasing the amplitude of the temperature vibration. The pulse number and the duty ratio do not affect the total input energy of the laser beam, but the temporal distribution of the laser energy is decided by the two parameters.

**Figure 8:**
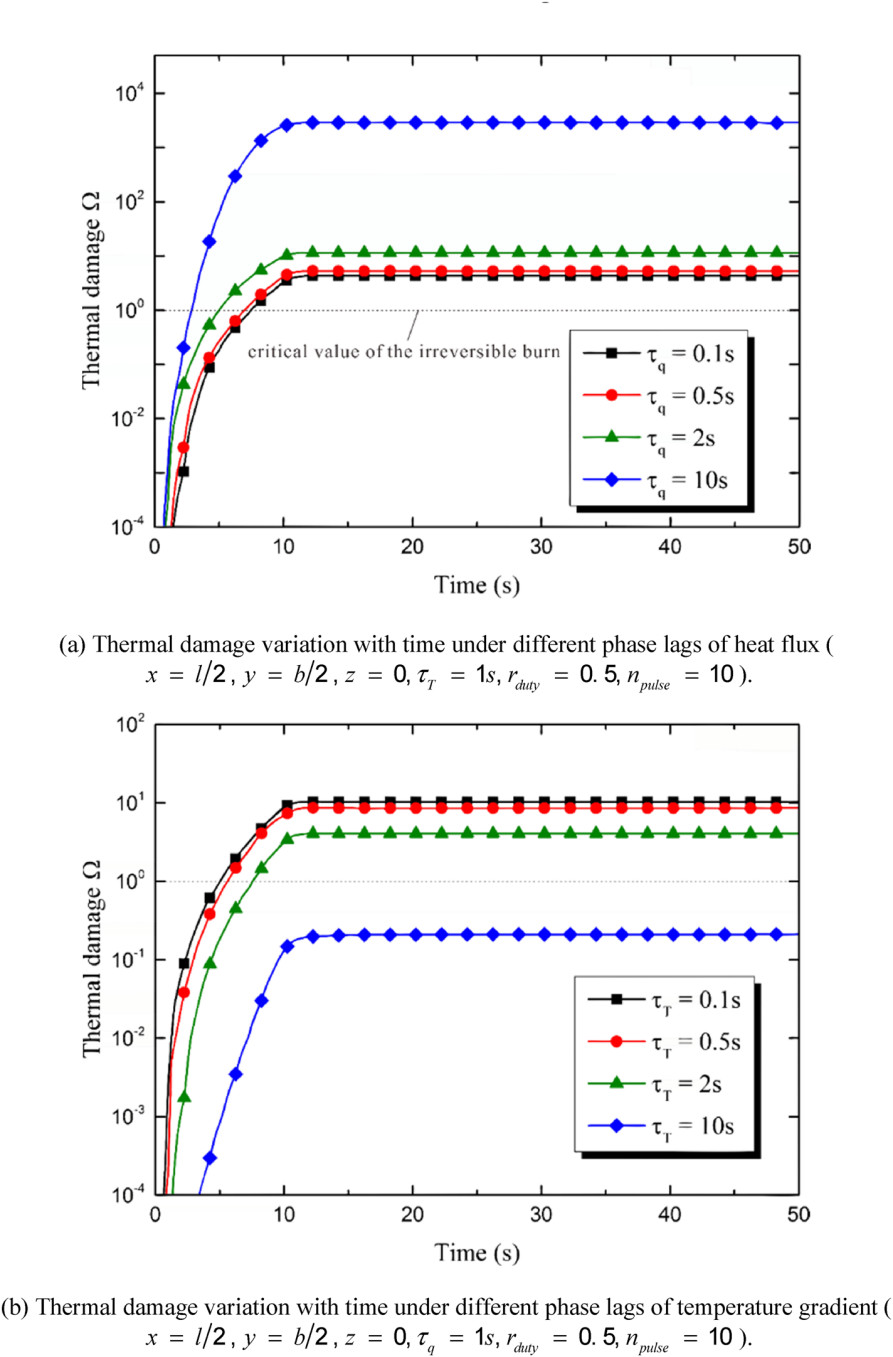
Influences of the phase lags on the thermal damage induced by the multi-pulse laser.

During the laser therapy, the skin is subjected to the laser pulses so that the temperature rise and the thermal damage occur in the irradiated region and the tumor cells will be killed. In order to protect the healthy part and kill the tumor efficiently, it is significant to estimate the burn degree in the irradiated skin. In the present study, the Henrique’s model^21^, which is based on the protein denaturation rate estimation, is adopted to assess the burn degree. Figure 8 shows the thermal damage variation with time at the center point of the irradiated area (*x* *=* *l*/2, *y* *=* *b*/2, *z* *=* 0). The parameters of the heat source are set to be *I*_*0*_ = 3 × 10^4^ W/m^3^, *r*_*duty*_ = 0.5, *n*_*pulse*_ = 10. The ordinate is set to be logarithmic to show the thermal damage more distinctly. Figure 8a shows the influences of the heat flux phase lag *τ*_*q*_. The phase lag of heat flux postpones the thermal balance progress and aggravates the heat accumulation. Consequently, more intense temperature rise will be induced, which has been shown in Fig. 6, and more severe burn is induced in the irradiated region. The value of the burn index Ω increases sharply during the heating stage (the former ten seconds) and then keeps constant after the heat source disappears. It is suggested that the thermal damage is irreversible when Ω reaches 1. Figure 8b shows that the influences of the temperature gradient phase lag *τ*_*T*_ is opposite to that of *τ*_*q*_. A great *τ*_*T*_ will promote the heat equilibrium process and reduce the heat accumulation in the irradiated region. As a result, the burn degree decreases with the increasing of *τ*_*T*_. It is shown in Fig. 8b that in the condition of *τ*_*q*_ = 1 s, no irreversible burn occurs in the skin when the temperature gradient phase lag increases to *τ*_*T*_ = 10 s.

**Figure 9:**
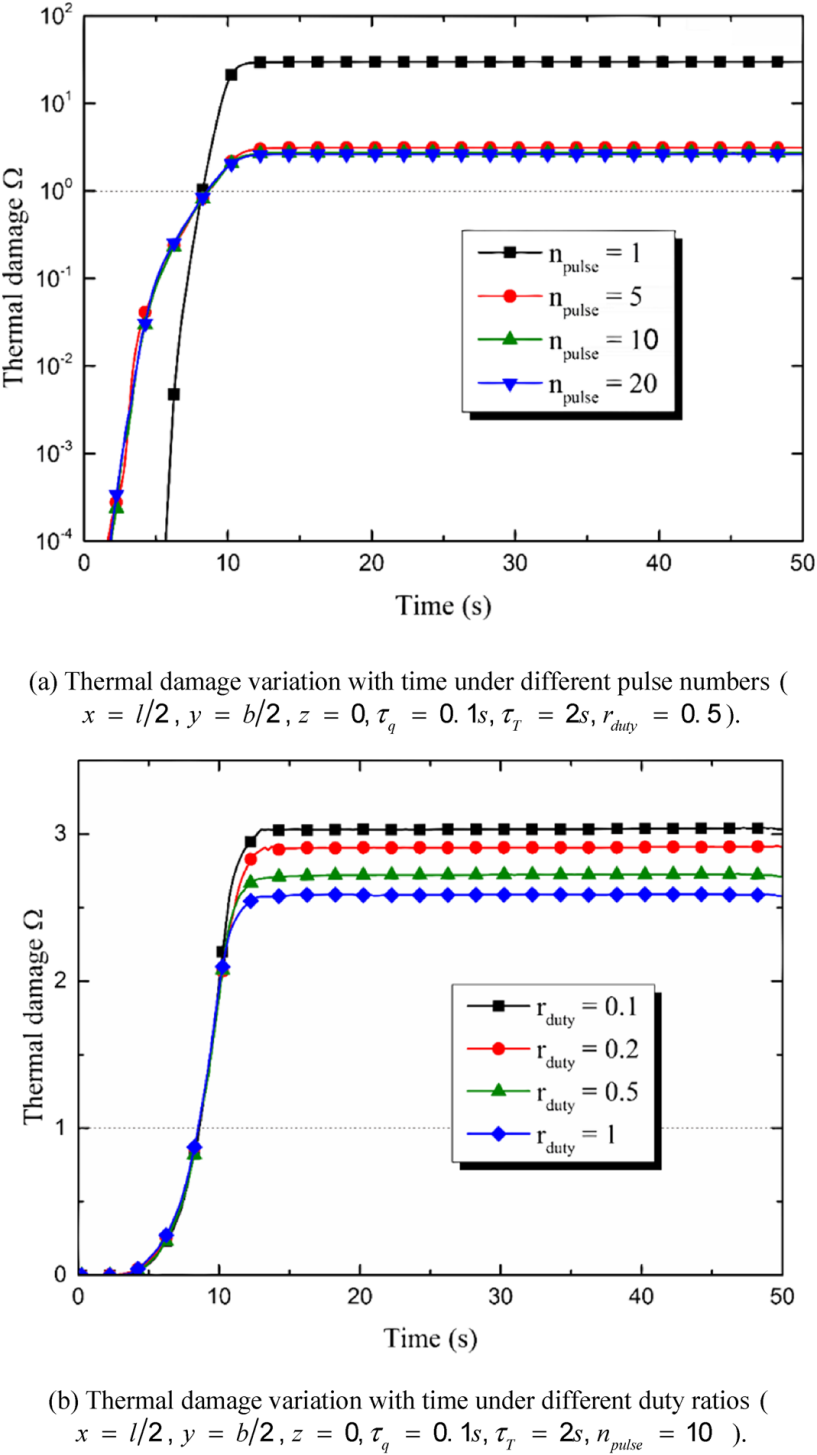

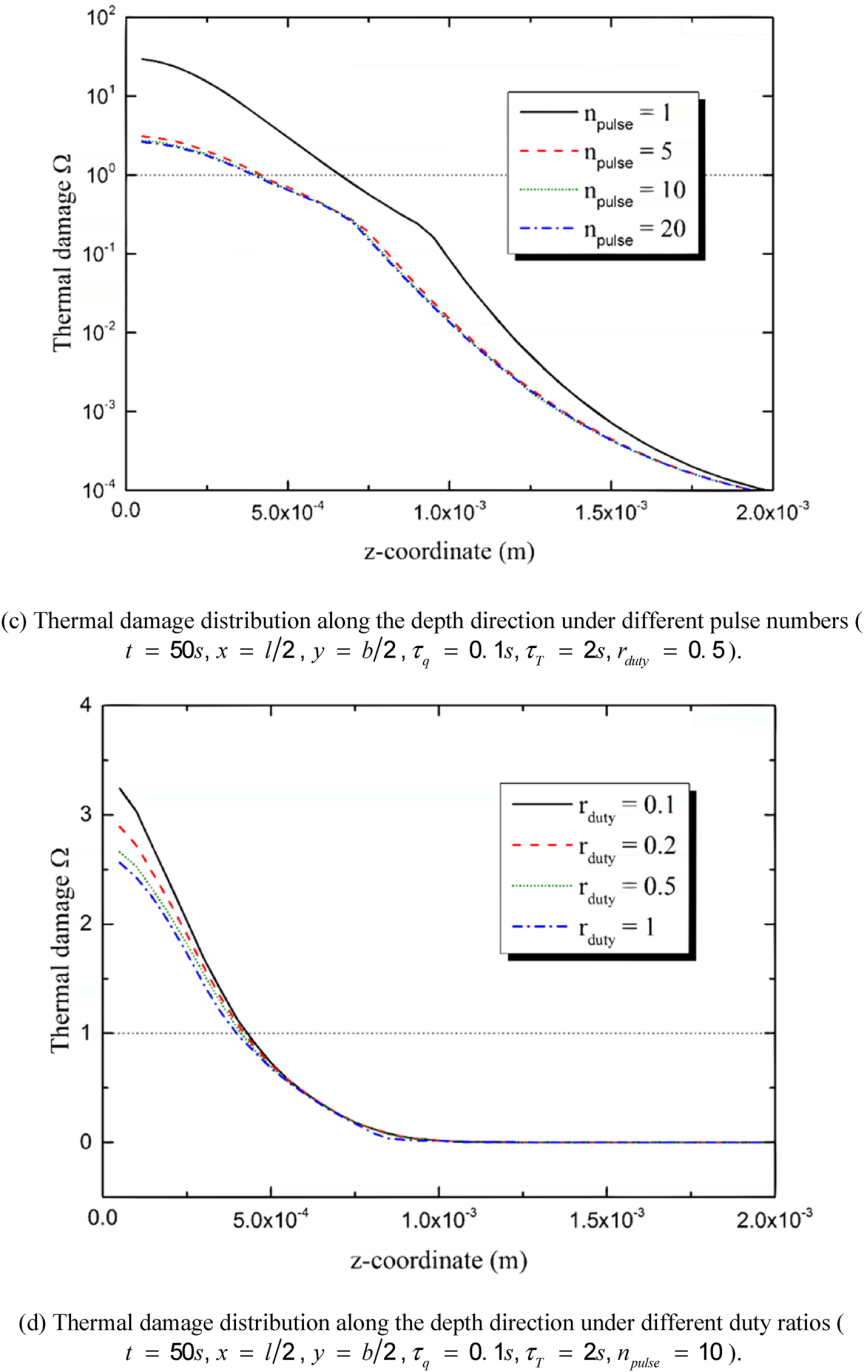
Influences of the heat source parameters on the thermal damage induced by the multi-pulse laser.

The parameters of the heat source, including the pulse number and the duty ratio, show crucial effects on the thermal response of the skin. As a result, the thermal damage in the irradiated region is also under the influences of these parameters. Figure 9a,b show the temporal distribution of the burn at the center point of the irradiated area. As is shown in Fig. 9a, the burn degree decreases with the increment of the pulse number *n*_*pulse*_. This tendency is tremendous especially when *n*_*pulse*_ ≤ 5. When *n*_*pulse*_ keeps increasing, the burn degree tends to that caused by a constant heat source. Figure 9b shows the influences of the duty ratio on the thermal damage. Under the premise of the constant energy input, the increment of the duty ratio results in the decrement of the burn degree. The obvious difference caused by duty ratio shows up at the end of the heating stage (i.e., the moment *t* *=* 10 s). Figure 9c,d show the thermal damage distribution along the depth direction under different pulse numbers and duty ratios, respectively. The burn occurs mainly in the small region close to the top surface of the skin. The thermal damage maximizes at the top surface and decreases with the increasing of the duty ratio and the pulse number. However, the comparison of Fig. 9a,c and b,d shows that the influences on the thermal damage caused by the pulse number are much more remarkable than that caused by the duty ratio. The area of region where is irreversibly burnt increases obviously when the value of *n*_*pulse*_ decreases from 5 to 1.

**Figure 10:**
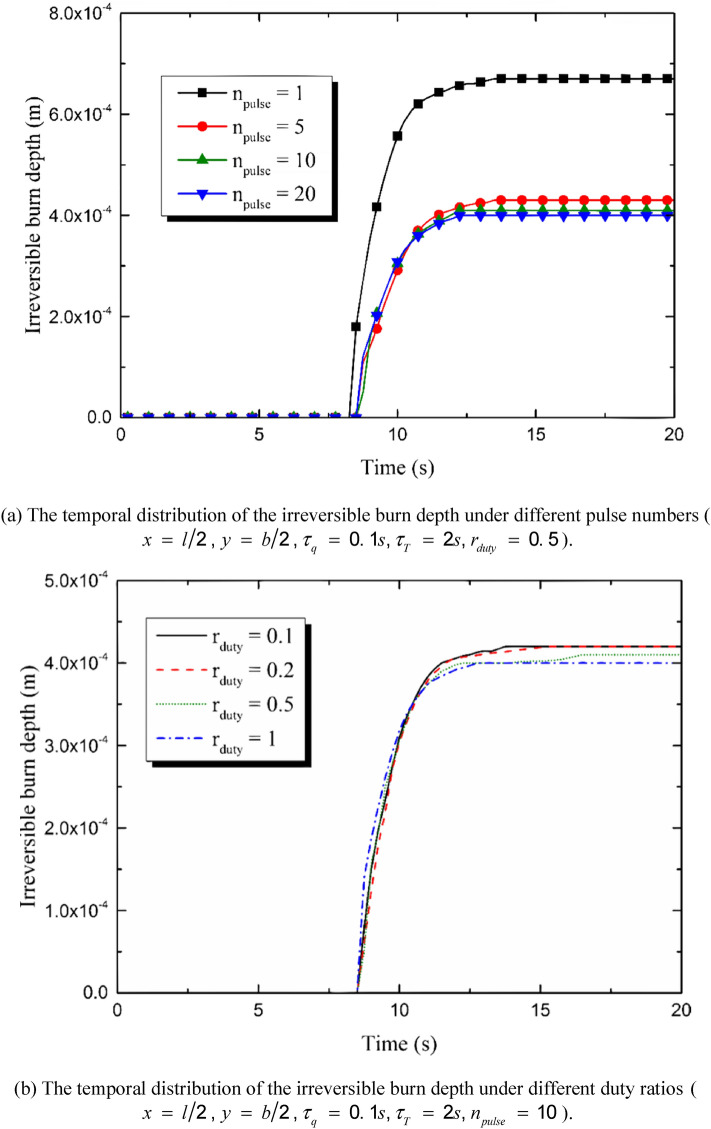

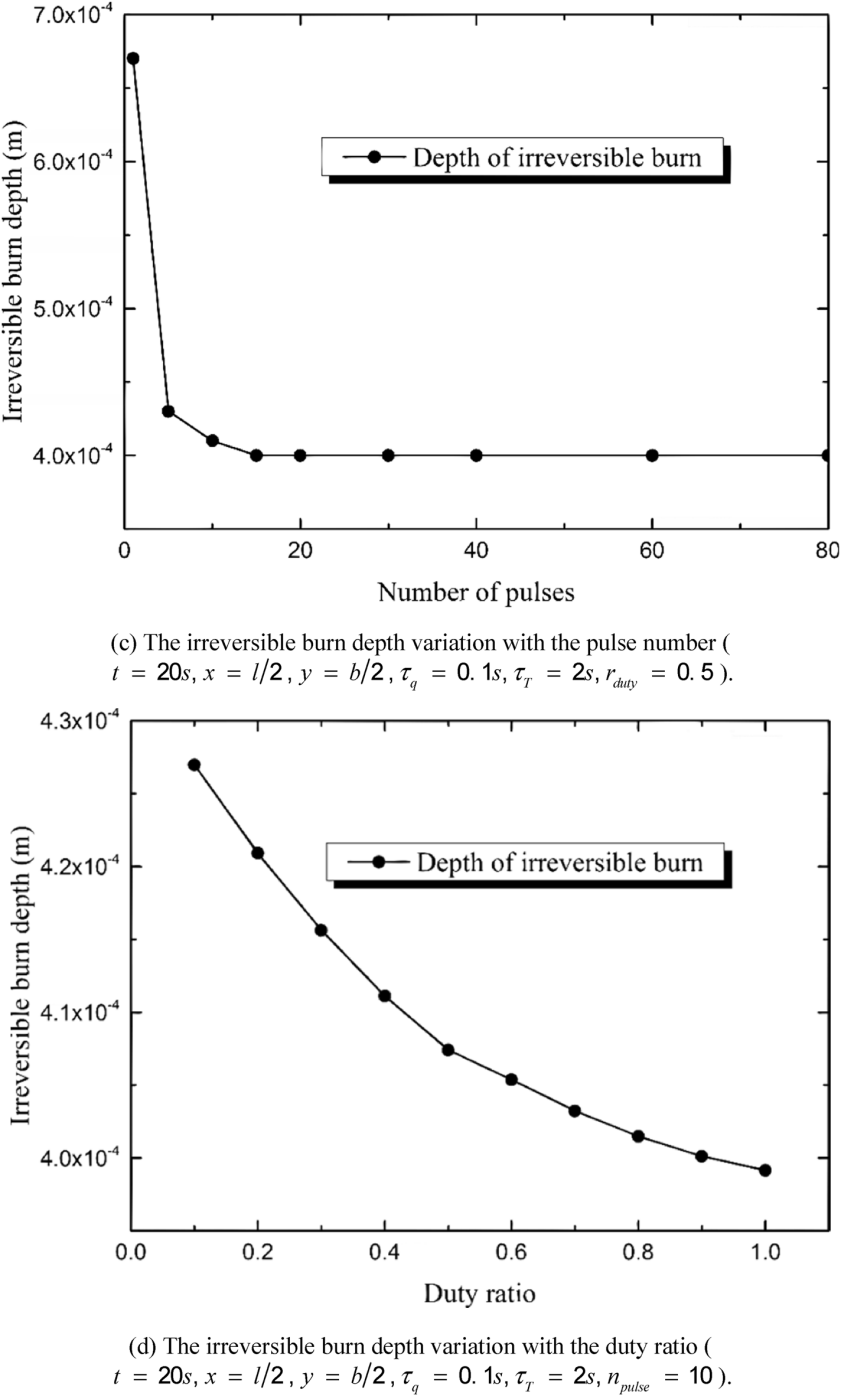
Influences of heat source parameters on the depth of the irreversible burn induced by the multi-pulse laser.

According to the Henrique’s model, the thermal damage is regarded to be irreversible when Ω reaches 1. In order to describe the irreversibly burnt region more clearly, the irreversible burn depth at the center point of the irradiated region has been calculated and the influences of the duty ratio and the pulse number are as shown in Fig. 10. Figure 10a,b show the time history of the depth. The irreversible thermal damage appears at the moment *t* *=* 7 s, extending downward for about eight seconds and then trends to be stable at the moment *t* *=* 15 s. The decrement of the pulse number leads to an increasing burn depth and takes a shorter time for the irreversible burn to appear, especially when *n*_*pulse*_ ≤ 5. The decreasing of the duty ratio also benefits the thermal damage. However, the appearing moment of the irreversible thermal damage changes little when the value of *r*_*duty*_ increases from 0.1 to 1. Figure 10c,d show the irreversible burn depth variation with the pulse number and the duty ratio, respectively. The burn depth reduces sharply when the pulse number increases from 1 to 5 and then tends to be stable with the continuous increment of pulse number. That is to say, on the premise of the constant input energy, single-pulse heat source causes the much deeper burn than multi-pulse does. The increasing of the duty ratio also leads to a decrement of the irreversible burn depth. However, the influence of the pulse number is more important than that caused by the duty ratio.

## Conclusions

The temperature response and thermal damage of skin were derived when it is subjected to repetitive pulse laser based on the DPL biological heat transfer model and the Henrique’s burn assessment model.

In the present study, the influences of the two phase lags and the heat source parameters on the temperature distribution and the thermal damage in the skin were researched. When *τ*_*q*_ is great, the temperature rises rapidly in the initial period of the heating stage and the rise tendency of the temperature gradually disappears as time passes. In addition, the increment of *τ*_*q*_ also leads to the increasing of the maximum temperature and the thermal damage in the irradiated region. On the contrary, the increasing of *τ*_*T*_ results in the decrement of the maximum temperature and the burn degree.

The duty ratio and the pulse number obviously affect the thermal response of the skin. The quantity of the total input energy is set to be constant. That is to say, the values of *n*_*pulse*_ and *r*_*duty*_ do not affect the quantity of the energy input but the energy temporal distribution. The peak temperature maximizes when *n*_*pulse*_ = 1 and decreases obviously with the increment of n_pulse_. As a result, the burn degree and the depth of the irreversible thermal damage decreases with the increment of *n*_*pulse*_. The influence of the pulse number is the most obvious when *n*_*pulse*_ = 1, keeping decreasing with the increment of *n*_*pulse*_ and gradually disappearing when *n*_*pulse*_ is great enough (*n*_*pulse*_ ≥ 50 in the calculation for example). The duty ratio has similar effects on the skin thermal response with the pulse number does. The increment of *r*_*duty*_ causes the decrement of the peak temperature, the burn degree and the depth of irreversible burn. However, the influences of the pulse number is more obvious than the duty ratio.

The validation of thermal damage parameter seems difficult because the quantitative description and measure is hard. However the temperature result validation is possible.
